# Long-Term Humoral Immune Response against SARS-CoV-2 after Natural Infection and Subsequent Vaccination According to WHO International Binding Antibody Units (BAU/mL)

**DOI:** 10.3390/v13122336

**Published:** 2021-11-23

**Authors:** Natalia Ruetalo, Bertram Flehmig, Michael Schindler, Lutz Pridzun, Angelika Haage, Marija Reichenbächer, Thomas Kirchner, Teresa Kirchner, Karin Klingel, Michael B. Ranke, Andrea Normann

**Affiliations:** 1 Institute for Medical Virology and Epidemiology, University Hospital Tuebingen, Elfriede-Aulhorn-Str. 6, 72076 Tuebingen, Germany; Natalia.Ruetalo-Buschinger@med.uni-tuebingen.de (N.R.); Michael.Schindler@med.uni-tuebingen.de (M.S.); 2 Paediatric Endocrinology, University Children’s Hospital, Hoppe-Seyler-Str. 1, 72076 Tuebingen, Germany; michael.ranke@gmx.de; 3 Mediagnost Gesellschaft für Forschung und Herstellung von Diagnostika GmbH, Aspenhaustr. 25, 72770 Reutlingen, Germany; Pridzun@mediagnost.de (L.P.); Haage@mediagnost.de (A.H.); Reichenbaecher@mediagnost.de (M.R.); 4 Pediatric Practice, Kapuzinerberg 17, 71263 Weil der Stadt, Germany; kirchner@kirchner-backhaus.de (T.K.); thk.tue@gmail.com (T.K.); 5 Institute for Pathology and Neuropathology, University Hospital Tuebingen, Liebermeisterstr. 8, 72076 Tuebingen, Germany; Karin.Klingel@med.uni-tuebingen.de

**Keywords:** COVID-19, SARS-CoV-2, anti-SARS-CoV-2 antibodies, SARS-CoV-2 S1-RBD-protein, SARS-CoV-2 N-protein, SARS-CoV-2 WHO standard, BAU

## Abstract

The new WHO reference standard allows for the definition of serum antibodies against various SARS-CoV-2 antigens in terms of binding antibody units (BAU/mL) and thus to compare the results of different ELISA systems. In this study, the concentration of antibodies (ABs) against both the S- and the N-protein of SARS-CoV-2 as well as serum neutralization activity were evaluated in three patients after a mild course of COVID-19. Serum samples were collected frequently during a period of over one year. Furthermore, in two individuals, the effects of an additional vaccination with a mRNA vaccine containing the S1-RBD sequence on these antibodies were examined. After natural infection, the antibodies (IgA, IgG) against the S1-protein remained elevated above the established cut-off to positivity (S-IgA 60 BAU/mL and S-IgG 50 BAU/mL, respectively) for over a year in all patients, while this was not the case for ABs against the N-protein (cut-off N-IgG 40 BAU/mL, N-IgA 256 BAU/mL). Sera from all patients retained the ability to neutralize SARS-CoV-2 for more than a year. Vaccination resulted in a rapid boost of antibodies to S1-protein but, as expected, not to the N-protein. Most likely, the wide use of the WHO reference preparation will be very useful in determining the individual immune status of patients after an infection with SARS-CoV-2 or after vaccination.

## 1. Introduction

So far, the detection of the humoral immune response after a SARS-CoV-2 infection has mainly been based on immunoassay systems measuring antibodies (ABs). Usually, these ABs are directed against the SARS-CoV-2 surface S1-protein, the so-called spike protein, and the inner nucleocapsid protein N of the virus. Most often, such assays measure antibodies of the classes IgG, IgA, and IgM. Until now, it has been difficult to compare the results obtained by different assay systems since the quantification of the viral ABs is not standardized. To solve this problem and to facilitate the comparison among different serological tests, the World Health Organization (WHO) has recently established and distributed reference material based on a standard serum pool collected from human donors having suffered from COVID-19 [1]. In this standard preparation, the quantity of antibodies is defined as 1000 neutralization antibody units (IU) corresponding to 1000 binding antibody units (BAU) per mL. Thus, it is now possible to compare and harmonize different commercially available test systems as well as clinical and serological studies.

The neutralizing activity of antibodies to SARS-CoV-2 is associated with IgG and IgA class antibodies directed against the receptor binding domain (RBD) of the spike protein [2,3,4,5]. Nevertheless, which quantities of antibodies reflect a valid protection against an infection with SARS-CoV-2 and/or against a COVID-19 disease are still poorly explored. Hence, it is of utmost importance to define the humoral immune status of individuals based on measurements standardized with the now available WHO reference. In addition, the effectiveness of the immune response after vaccination with the various established vaccines can now be compared.

We have developed highly specific ELISA systems for the detection of IgG and IgA class antibodies directed to the spike (RBD) protein as well as to the nucleoprotein (N) of SARS-CoV-2 [6]. By applying these test systems, the above-mentioned WHO standard was evaluated and used to measure the immune response of patients after a natural infection with SARS-CoV-2.

## 2. Materials and Methods

### 2.1. Ethics

Experiments conformed to the principles of the Declaration of Helsinki. Written informed consent was obtained from all blood donors prior to the study. The study was approved by the Independent Research Ethics Committee (IEC) of the University of Tuebingen, IEC-Project Number 672/2020A, 2020-08-19.

### 2.2. Patients

All patients were related and lived in the same household. Patient 1: Adult male, 53 years old. The first symptom was coughing, lasting until day 8 after onset of disease. Fever (38 °C) on days 2 and 3. Symptoms on days 2–8 included headache, muscle pain in arms and legs, and fatigue. Apart from occasional coughing after day 8, the patient’s well-being improved continuously until full recovery of health was reached. Patient 2: Adult female, 52 years old. The first symptom, coughing, occurred three days after the onset of disease in patient 1. On days 4 and 5, body temperature was elevated (37–38 °C). Headache and limb pain were noted on days 2–8. Persistent tiredness started on day 4, anosmia occurred from day 5–13. Subsequently, the patient recovered fully. Patient 3: Adult female, 22 years old. Three days after patient 1, she began coughing, had elevated temperature (37–38 °C) on days 2 and 3, muscle pain on day 3, rhinitis on days 5–7, and hyposmia on days 2–8. She was fully recovered by day 8.

In all patients, SARS-CoV-2 RNA had been diagnosed via nasal and oro-pharyngeal fluids. Testing was conducted in a commercial diagnostic laboratory on day 1, when the first symptoms occurred. The first nasal swab was not quantified; however, RNA could be quantitatively detected in follow-up testing over a period of four weeks in all three patients [6].

In our study, blood samples were taken from patients for over a year at the following time intervals. Patient 1: on days 13, 20, 25, 33, 40, 48, 60, 74, 109, 137, 165, 193, 228, 246, 299, 326, 355, 392, 417, and 452 after onset of symptoms; Patient 2: on days 10, 17, 22, 30, 37, 45, 57, 71, 106, 134, 162, 190, 225, 243, 296, 323, 352, 389, 414, and 449 after experiencing first symptoms; Patient 3: on days 11, 18, 23, 31, 38, 46, 58, 72, 107, 135, 163, 191, 226, 244, 297, 324, 381, and 423 days after the occurrence of symptoms.

Patient 1 and patient 2 received a mRNA vaccine (BNT162b2 vaccine from Pfizer/BioNTech) containing the sequence of the S1-protein between days 392 and 417 (patient 1) and between days 389 and 414 (patient 2), in other words, after the acute phase of their COVID-19 disease.

### 2.3. Detection of IgA and IgG Antibodies against SARS-CoV-2-S1-RBD and Nucleoprotein

IgA and IgG antibody detection directed to the S1-RBD SARS-CoV-2 in human sera using E111-IVD (anti-SARS-CoV-2 IgG ELISA, Mediagnost GmbH, Reutlingen, Germany) and E113 (anti-SARS-CoV-2 IgA ELISA, Mediagnost GmbH, Reutlingen, Germany) were performed according to the manufacturer’s instructions. These have been described in detail earlier [6,7]. The IgA and IgG ELISA for the determination of antibodies directed against the SARS-CoV-2 nucleoprotein (N-Protein) in human sera were developed and performed in analogy to the assays for anti- S1-RBD SARS-CoV-2. They were also described in detail earlier [6,7].

### 2.4. Calibration with the WHO Reference Material

The first WHO International Reference panel of anti-SARS-CoV-2 immunoglobulin consisted of pooled plasma samples from individuals recovered from COVID-19 disease and a negative control plasma obtained from healthy blood donors before 2019. The panel was evaluated in a WHO international collaborative study [1].

The standard is distributed in a freeze-dried manner delivered by the National Institute for Biological Standards and Control (NIBSC)-code 20/136 and is described in detail [1]. The unitage and assigned potency of this standard for SARS-CoV-2 is in the final concentration after reconstitution of 1000 IU per mL for neutralizing antibody activity. For binding antibody assays, an arbitrary unitage of 1000 binding antibody units per mL (BAU/mL) can be used to compare assays detecting the same class of immunoglobulins with the same specificity [1].

The modification for the quantitative detection of these antibodies was made as follows. A calibration reagent CAL IS-20/136 containing 2.5 binding antibody units (BAU) was used. This internal calibration reagent was prepared with the serum from a recovered COVID-19 patient and defined for its anti-SARS-CoV-2 S1-RBD IgG-content and its anti-SARS-CoV-2-S1-RBD IgA-content in comparison to the WHO COVID-19 standard NIBSC 20/136 [1]. This internal reference is known for its neutralizing activity.

The calibration reagent used, CAL-IS-20/136, contains different BAU/mL anti-SARS-CoV-2 S1-RBD contents: 2.5 BAU, 1.25 BAU, 0.625 BAU, 0.313 BAU, 0.156 BAU, 0.0878 BAU, and used as standard dilutions in the anti-SARS-CoV-2 ELISA E111. For all other assays, the procedure was analogous. Using a statistical algorithm, a standard curve was calculated by which the unknown samples could be quantified. Based on our preliminary results, antibody levels above 50 BAU/mL were defined as positive.

The cut-off value between negative and positive samples was calculated by OD values of negative samples multiplied by five. OD values greater than five times the negative control were defined as positive (for calculations examples, see Supplementary Materials S1). Based on the statistical algorithm of the standard curve, the five times OD values of the negative control corresponded to about 50 BAU/mL. The WHO defines values for anti-SARS-CoV-2-S1-RBD IgG of about 44–53 BAU/mL as low titers, 200–300 BAU/mL as mid titers, and 700–800 BAU/mL as high titers. Thus, our cut-off value of 50 BAU/mL anti-SARS-CoV-2-S1-RBD IgG is in agreement with the low titer values published by WHO [1]. The cut-off value to positivity was found to be 60 BAU/mL for anti-SARS-CoV-2-S1-RBD IgA. By the same definition, the cut-off levels for anti-SARS-CoV-2 nucleoprotein IgA were defined as 250 BAU/mL and for anti-SARS-CoV-2 nucleoprotein IgG 40 BAU/mL.

### 2.5. Virus Neutralization Assay (VNA)

The SARS-CoV-2 strain icSARS-CoV-2-mNG [6,7] was obtained from the World Reference Center for Emerging Viruses and Arboviruses (WRCEVA) of the UTMB (University of Texas Medical Branch, Galveston, TX, USA). To generate icSARS-CoV-2-mNG stocks, Caco-2 cells were infected, the supernatant was harvested 48 h post-infection, centrifuged, and stored at −80 °C. For MOI (multiplicity of infection) determination, a titration using serial dilutions of the virus stock was conducted. The number of infectious virus particles per mL was calculated as the (MOI  ×  cell number)/(infection volume), where MOI  =  −ln (1 − infection rate).

Caco-2 (human colorectal adenocarcinoma) cells were cultured at 37 °C with 5% CO_2_ in DMEM (Dulbecco’s modified Eagle medium) containing 10% FBS (fetal bovine serum), with 2 mM L-glutamine, 100 ug/mL penicillin-streptomycin, and 1% NEAA (non-essential amino acids).

For neutralization experiments, 1 × 10^4^ Caco-2 cells/well were seeded in 96-well plates the day before infection, in media containing 5% FBS. Cells were infected with the SARS-CoV-2 strain icSARS-CoV-2-mNG at a MOI = 0.3 or mock-infected. Immediately after infection, serum from our three patients was added to each well in two-fold serial dilutions from 1:40 up to 1:5120. The cells were fixed 48 h post-infection with 2% PFA (paraformaldehyde) and stained with Hoechst 33,342 (1 µg/mL final concentration) for 10 min at 37 °C. The staining and fixing solutions were removed and rinsed with PBS. In order to quantify the infection rates, images were taken with the Cytation3 (Biotek), and mNG+ cells were automatically counted by using the Gen5 software (Biotek). Titers of neutralizing antibodies (NAbs) were calculated as the half-maximal inhibitory dose (i.e., the virus neutralizing titer 50 (VNT50)) using 4-parameter nonlinear regression (GraphPad Prism). In order to obtain VNT50 values comparable to the data already published in Flehmig et al. (2020), the VNT50s were normalized according to the MOIs used in each experiment.

## 3. Results

Serum samples were collected from three patients over a period of about one year after the acute phase of COVID-19 and were analyzed for IgA and IgG antibodies against the S1-protein as well as N-protein. The quantity of antibodies was determined by using the newly developed WHO standard as described above.

**Figure 1:**
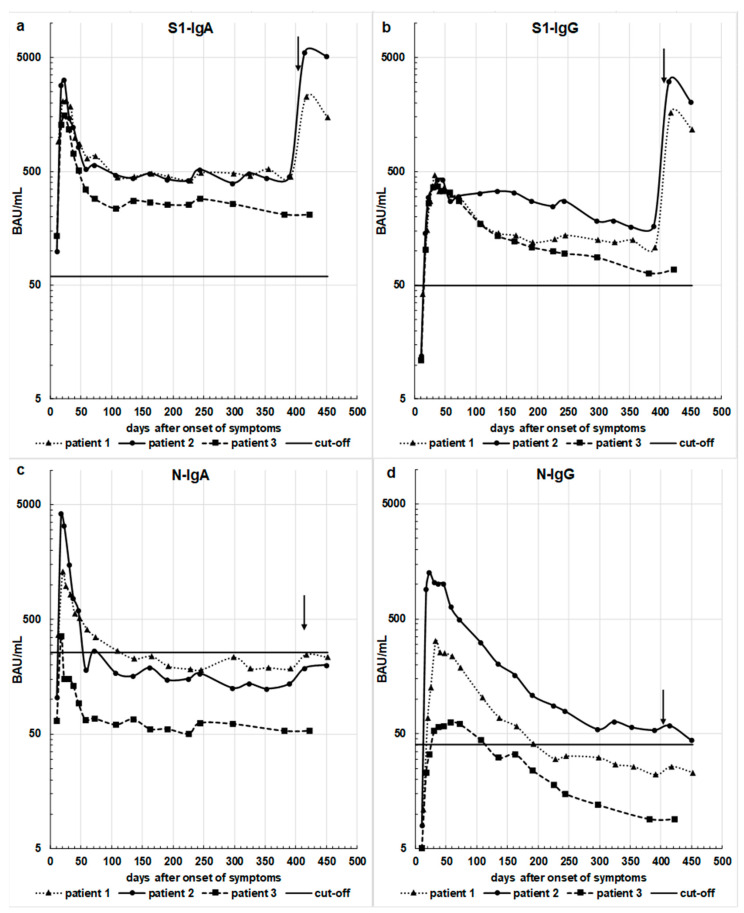
Concentrations of antibodies against SARS-CoV-2 S-protein (**a**): S1-IgA-(cut-off: 60 BAU/mL); (**b**): S1-IgG- (cut-off: 50 BAU/mL) and antibodies against SARS-CoV-2 N-protein (**c**): N-IgA-(cut-off: 250 BAU/mL); (**d**): N-IgG- (cut-off: 40 BAU/mL) expressed in terms of WHO BAU/mL in individual patients related to time [patient 1: day 13–452, patient 2: day 10–449, patient 3: day 11–423]) after the clinical beginning of COVID-19 disease. The vertical arrows in all figures mark the day of vaccination of patients 1 and 2, (BNT162b2 vaccine from Pfizer/BioNTech). The horizontal lines mark the respective cut-off levels.

Figure 1a–d illustrates the course of serum antibodies over time IgA and IgG directed against the S1- or the N-protein.

The course of IgA antibodies directed against S1 or N shows a similar pattern until vaccination. After infection, there was a steep rise with the peaks occurring around 20 to 24 days after onset of symptoms. Thereafter, a moderate decline until 60 to 73 days after onset of symptoms was observed, followed by a very slow decline during the months afterward. The anti-S1 IgA class antibodies were measured at the same time, roughly one year after the acute phase of their COVID-19 disease. The results were 457 BAU/mL, 481 BAU/mL, and 210 BAU/mL for patients 1, 2, and 3, respectively. The levels were all above the cut-off level of 60 BAU/mL for S1-IgA. While all levels of S1-IgA stayed above the cut-off to positivity, this was not the case for N-IgA in any of the patients.

The course of the IgG levels against both the S1- or the N-protein resembled each other but differed from that of the IgA levels. The rise to a maximum around 24 days after onset of symptoms was equally steep, reaching a maximum about 47 days after onset of symptoms. Subsequently, there was a very protracted decline lasting about one year after onset of symptoms. Remarkably, all anti N-IgG levels reached or even declined below the cut-off of positivity by the end of the observation phase, while this was not the case for anti S1-IgG. In addition, while antibodies of the IgG class against the N protein declined below the cut-off level in two out of the three patients between 100 and 200 days after symptom onset, the IgA antibodies fell below the cut-off level in all three patients already at about 100 days after the onset of the disease. As current vaccines are designed to express the S-protein as an immunogen, we observed a steep rise in IgA and IgG levels against S1, but not the N-protein after vaccination.

**Figure 2:**
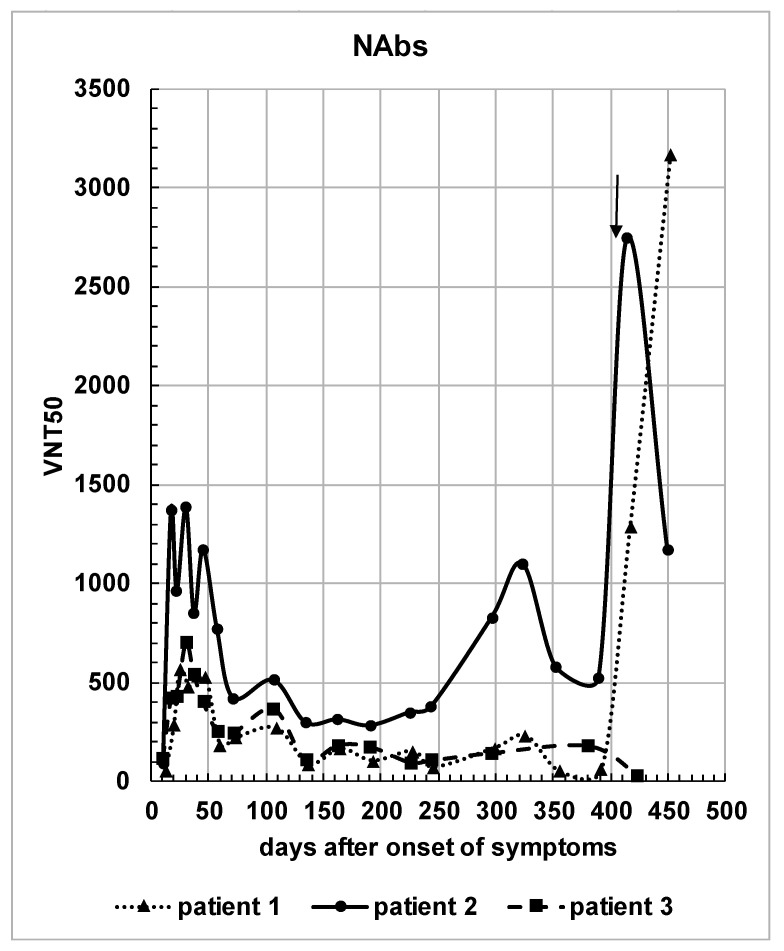
Serum virus neutralizing activity of the individual patients over time. VNT50s (serum dilution inhibiting the half-maximal infection) are plotted against days after symptom onset. The arrow indicates the time of immunization for patients 1 and 2.

The course of NAbs for the three patients is illustrated in Figure 2. In contrast to the courses of IgA and IgG directed against S1- or N-protein, the levels of NAbs mainly followed the antibody response against the S1-protein, as expected. Marked virus-neutralizing activity was observed as early as day 20, which rose further until about day 40. However, a decline followed and markedly lower, yet positive levels, were recorded over time. Interestingly, at day 320, there was another increase of NAbs in patient 1 (mild increase) and patient 2 (moderate increase), which is inexplicable.

Patients 1 and 2 received a vaccination with the mRNA vaccine encoding for the sequence of the S1-protein from BioNTech about 12 months after the acute phase of their COVID-19 disease. The vaccination elicited a strong antibody boost effect with a very high increase in VNT50 values for patients 1 and 2. The significant increase in the neutralization ability of sera from patients 1 and 2 correlated with the steep increase in both S1-IgA and S1-IgG antibody levels (Figure 1a,b).

## 4. Discussion

It is common practice to detect SARS-CoV-2 infection by measuring SARS-CoV-2 RNA genome equivalents using RT-PCR from material taken from the oropharynx [8,9]. The patients’ immune response can be measured by specific antibodies against various viral proteins by proving virus-specific neutralizing activities and/or by evaluating the cellular immunity [8]. However, the latter two methods are complex and therefore not suited for diagnostic purposes or for examining a large number of patient samples.

Measuring antibodies in blood samples with ELISA systems is a simpler and therefore more practical approach. We and others have shown [6,7,8,10,11,12,13] that binding antibodies as well as neutralizing antibodies directed against the S-protein of SARS-CoV-2 persist over a period of about half a year. So far, most of the published antibody detection systems describe the quantity of detectable antibodies in human sera in a semiquantitative manner. However, a more profound understanding of the humoral immune response to a virus infection such as SARS-CoV-2 requires a quantitative evaluation of the antibody response. Due to the relatively short time that has passed since the beginning of the COVID-19 pandemic, information about the long-term antibody response in patients is still limited. Recent reports, however, suggest that patients who recovered after COVID-19 disease are protected against severe illness after reinfection for at least one year [14,15,16].

In this study, we followed the development of antibodies against SARS-CoV-2 S- and N-protein for more than one year, using a small group of patients. While we previously monitored antibody development over a course of six months with a semiquantitative ELISA [6], we have now used the new WHO standard for the quantitative detection of antibodies against SARS-CoV-2 [1]. In addition, the immune response of patients 1 and 2 following mRNA vaccination, roughly one year after their natural SARS-CoV-2 infection, is shown. We observed that after a natural infection, the antibody levels declined progressively over time, and that the vaccination in our patients resulted in a remarkable quantitative increase in antibodies directed against the S1-protein, but not against the N-protein measured two weeks after vaccination. Our results are confirmed by other studies [17,18,19]. The huge boost in our patients is also reflected in the serum antibodies’ neutralizing activity. The transient increase in the NAbs before the vaccination of patient 1, and especially of patient 2 at day 320 (see Figure 2), is unexplained, but may be the result of an unknown exposure to SARS-CoV-2 or another cross-reacting coronavirus [20,21].

At present, it is not known how much of the antibodies directed to the S1- or N-protein reflect an immunity against SARS-CoV-2. Based on our data, we had assumed a level of 50 BAU per mL as a working cut-off level of positivity. A similar level was also reported when evaluating the new WHO International preparation (NIBCS code 20/268) for anti-SARS-CoV-2 immunoglobulin. Nevertheless, the biological relevance of this value has not been unequivocally established yet [22].

In virus infections eliciting a lifelong humoral antibody response such as in hepatitis A or measles, prevalence studies are easy to perform, since the disappearance of antibodies pose no or only minor problems of false negative results [23,24]. In cases in which a virus infection induces only a limited humoral immune response such as influenza, respiratory infections, or seasonal coronavirus infections, the diagnostic and epidemiological studies are more difficult to interpret [25]. Evaluating the dynamics of the antibody response after a natural infection is of great importance for dissecting the pathogenesis of a specific infection. In addition, knowing the continuity of the antibody response after an acute infection is highly relevant for recommending or abstaining from a revaccination. Thus, a standardized quantification of anti-SARS-CoV-2 antibodies in the blood is of utmost importance. A validated comparison between BAU/mL values and VNT (50) levels will allow in the future to harmonize results related to the humoral immune response against SARS-CoV-2 between different studies. Future studies must show how long measurable antibodies against SARS-CoV-2 persist and most importantly, which amounts of antibodies in terms of BAU protect against reinfection or against developing a severe COVID-19 disease.

## Data Availability

The datasets used and/or analyzed during the current study are available from the corresponding author upon reasonable request.

## Supplementary Materials

The following are available online at https://www.mdpi.com/article/10.3390/v13122336/s1, Figure S1: Mediagnost anti-Sl RBD lgA WHO 20/136 CAL-Curve
