# Supplementary material for: Long-Term Humoral Immune Response against SARS-CoV-2 after Natural Infection and Subsequent Vaccination According to WHO International Binding Antibody Units (BAU/mL)
Source: Viruses. 2021 Nov 23;13(12):2336. doi: 10.3390/v13122336 (PMC8708153; doi:10.3390/v13122336)

Mediagnost **anti-SARS-CoV-2 S1 RBD human IgA**
Exemplary response curve WHO NIBSC 20/136

Concentration 20/136 BAU/mL	Optical Density 450-620nm Blank corrected, arbitrary Units
0,000	0
0.078	0.316
0.156	0.599
0.312	1.049
0.625	1.712
1.250	2.544
2.500	3.474

		corresponding BAU/mL	
Negative Control (1:201dil.)	0.224	0.045	9.045
**5 x NC - cut off**	1.120	0.346	**69.55**

Curve Formula	Parameter	Value	Std. Error	95% CI min	95% CI max	
Y=D*X^3+C*X^2+B*X+A	A	0,0636	0,0278	-0,0561	0,183	
	B	3,6	0,149	2,96	4,24	
	C	-1,7	0,164	-2,4	-0,989	
	D	0,321	0,0442	0,13	0,511	
Curve Name	Curve Formula	A	B	C	D	R2
StdCurve	Y=D*X^3+C*X^2+B*X+A	0,0636	3,6	-1,7	0,321	1

Mediagnost anti-S1 RBD IgA
Test vom 15.06.2021, JE

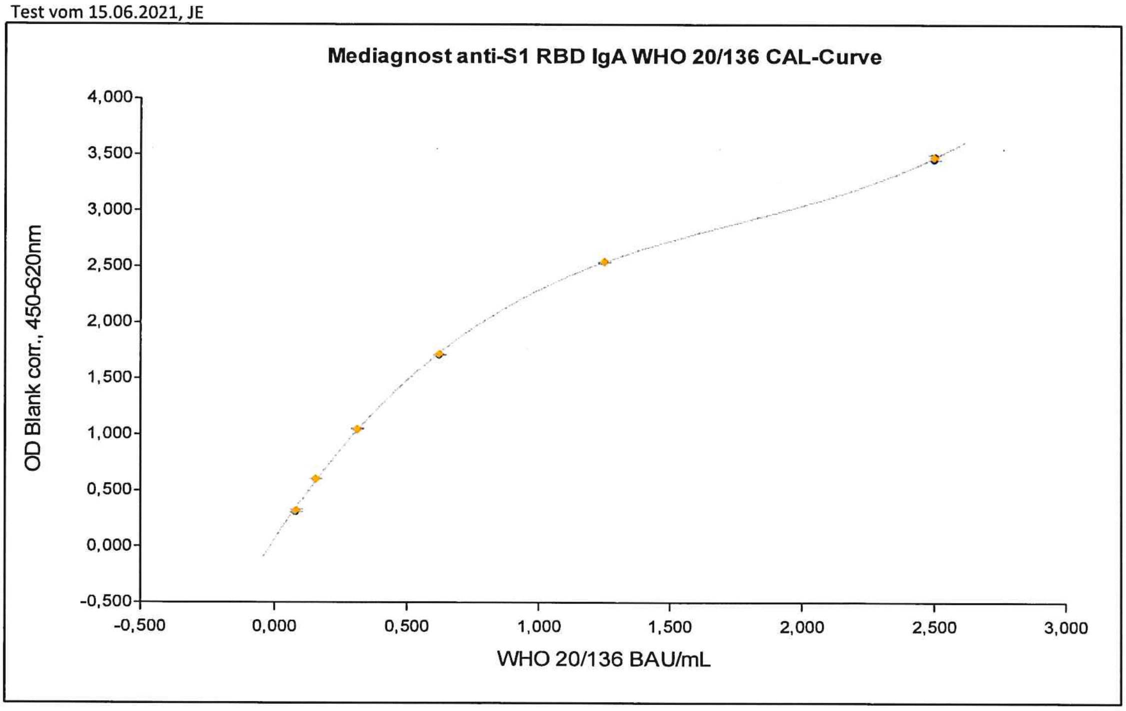

Supplemental Data - Manuscript ID: viruses-1369783
Mediagnost **anti-SARS-CoV-2 S1 RBD human IgG**
Exemplary response curve WHO NIBSC 20/136

Concentration 20/136 BAU/mL	Optical Density 450-620nm Blank corrected, arbitrary Units
0	0
0.078	0.193
0.156	0.384
0.312	0.735
0.625	1.310
1.250	1.971
2.500	2.698

		corresponding BAU/mL	calculated final conc. BAU/mL
Negative Control (1:201dil.)	0,124	0.050	10.045
**5 x NC - cut off**	0.620	0.255	**51.264**

Curve Formula	Parameter	Value	Std. Error	95% CI min	95% CI max
Y=D*X^3+C*X^2+B*X+A	A	-0,0116	0,00875	-0,0394	0,0163
	B	2,77	0,0539	2,6	2,94
	C	-1,22	0,0636	-1,42	-1,02
	D	0,218	0,0176	0,161	0,274
Curve Formula	A	B	C	D	R2
Y=D*X^3+C*X^2+B*X+A	-0,0116	2,77	-1,22	0,218	1

Mediagnost anti-S1 RBD IgG
Test vom 23.02.2021, MR

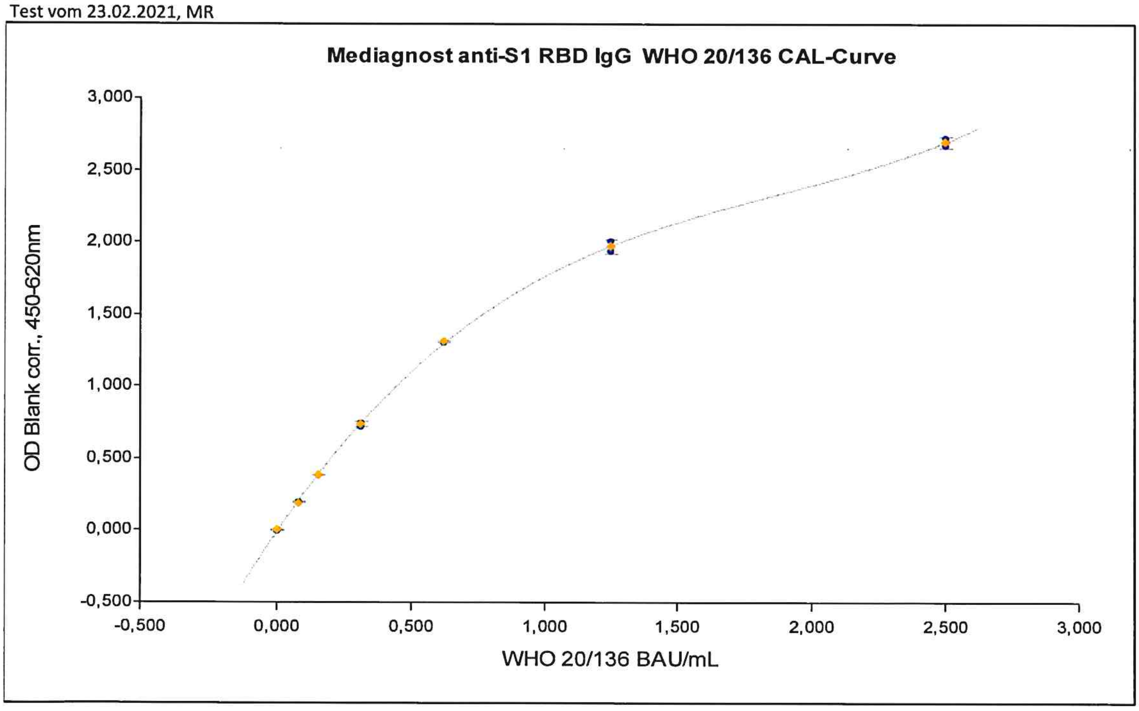

Mediagnost **anti-SARS-CoV-2 N human IgA**
Exemplary response curve WHO NIBSC 20/136

Concentration 20/136 BAU/mL	Optical Density 450-620nm Blank corrected, arbitrary Units
0,000	0
0.312	0.143
0.625	0.296
1.250	0.543
2.500	1.137
5.000	1.931
10.000	2.838

		corresponding BAU/mL	calculated final conc. BAU/mL
Negative Control (1:201dil.)	0.118	0.259	52.059
**5 x NC - cut off**	0.590	1.275	**256.275**

Curve Formula	Parameter	Value	Std. Error	95% CI min	95% CI max
Y=C*X^2+B*X+A	A	-0,00883	0,0164	-0,0545	0,0368
	B	0,496	0,0115	0,464	0,528
	C	-0,0212	0,00113	-0,0243	-0,0181

Curve Formula	A	B	C	R2
Y=C*X^2+B*X+A	-0,00883	0,496	-0,0212	1

Mediagnost anti-N IgA
Test vom 23.02.2021, MR

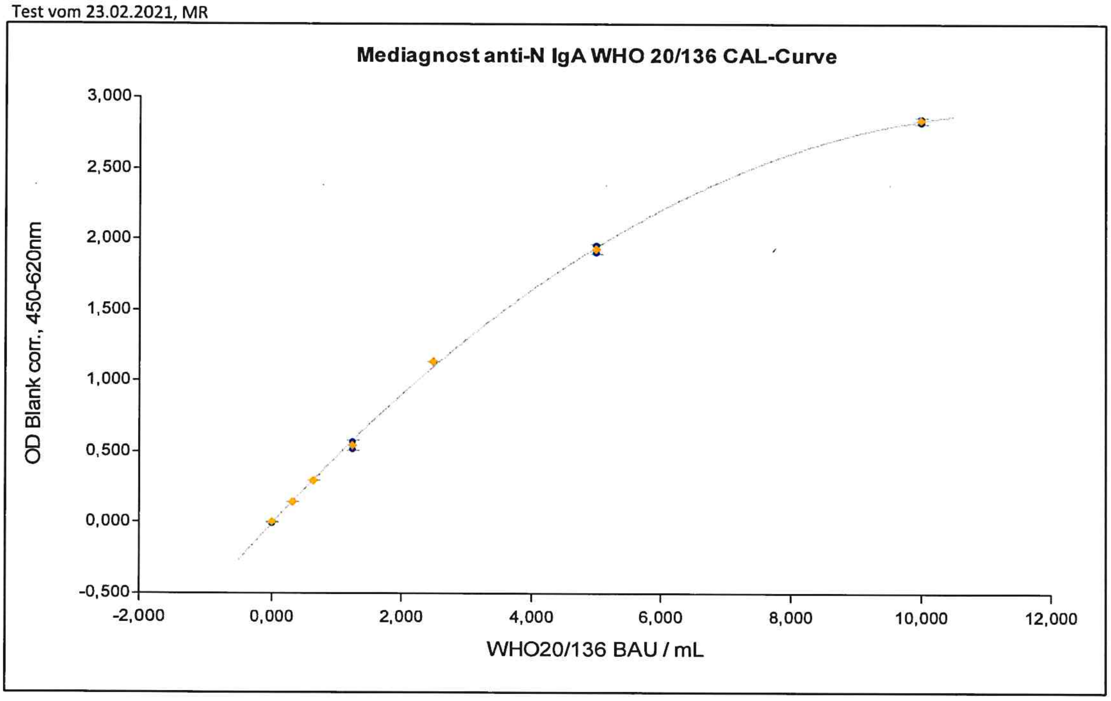

Supplemental Data - Manuscript ID: viruses-1369783
Mediagnost **anti-SARS-CoV-2 N human IgG**
Exemplary response curve WHO NIBSC 20/136

Concentration 20/136 BAU/mL	Optical Density 450-620nm Blank corrected, arbitrary Units
0	0
0.039	0.227
0.078	0.447
0.156	0.778
0.312	1.252
0.625	1.983

		corresponding BAU/mL	calculated final conc. BAU/mL
Negative Control (1:201dil.)	0.178	0.029	5.763
**5 x NC - cut off**	0.89	0.194	**39.027**

Curve Formula	Parameter	Value	Std. Error	95% CI min	95% CI max
Y=C*X^2+B*X+A	A	0,0382	0,0339	-0,0698	0,146
	B	4,97	0,345	3,87	6,07
	C	-2,99	0,528	-4,67	-1,31

Curve Formula	A	B	C	R2
Y=C*X^2+B*X+A	0,0382	4,97	-2,99	0,998

Mediagnost anti-N IgG
Test vom 24.02.2021, MR

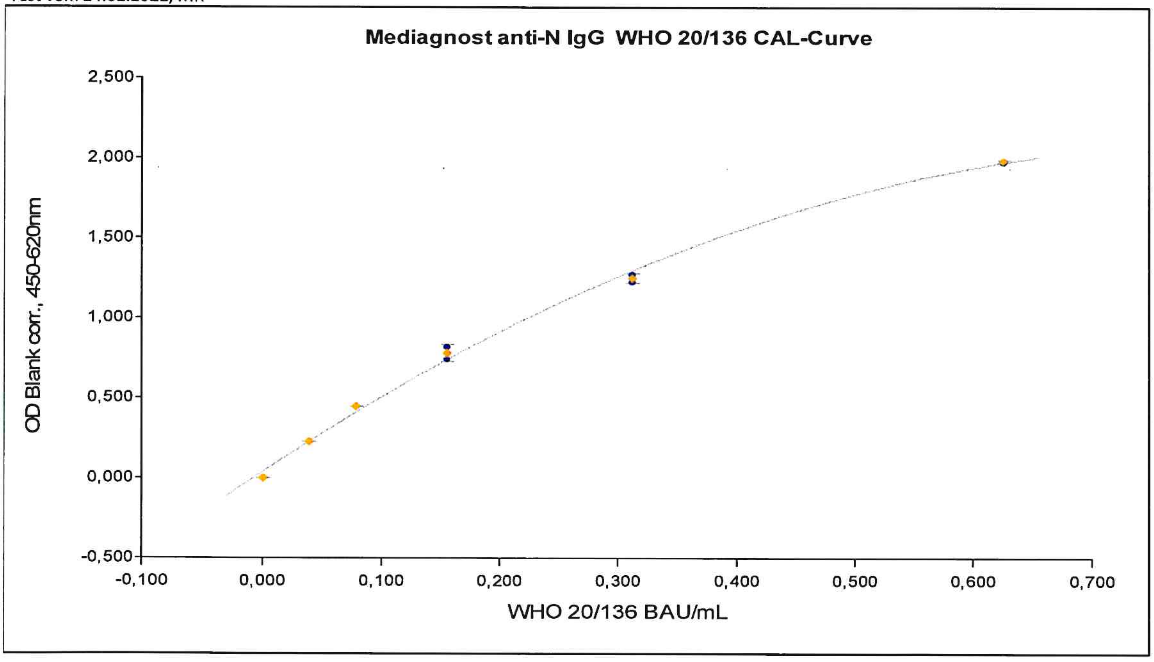

Supplement: Supplementary file 1 [file viruses-13-02336-s001.zip › viruses-1450273-supplementary.pdf]
